# Refractory Epistaxis due to Severe Factor V Deficiency with Inhibitor

**DOI:** 10.1155/2015/603402

**Published:** 2015-08-09

**Authors:** Elizabeth S. John, Minesh D. Patel, Julio Hajdenberg

**Affiliations:** ^1^Department of Medicine, Robert Wood Johnson Medical School, 125 Paterson Street, New Brunswick, NJ 08901, USA; ^2^University of Central Florida, 4000 Central Florida Boulevard, Orlando, FL 32816, USA; ^3^Division of Hematology and Oncology, University of Florida Health Cancer Center in Orlando, 1400 S Orange Avenue, MP700, Orlando, FL 32806, USA

## Abstract

Factor V deficiency secondary to inhibitors is extremely rare and can be caused by a wide collection of exposures such as bovine thrombin and beta lactamase antibiotics. The management of factor V deficiency with inhibitor is a condition treated based on case reports due to the rarity of this condition. We describe a complicated case of an elderly patient with severe factor V deficiency with high inhibitor titer refractory to FEIBA (anti-inhibitor coagulation complex) treated with NovoSeven concurrently with cyclosporine immunosuppression and Rituxan. Given that there are no consensus guidelines on treatment, this case offers important insight into the therapeutic approaches that can be used to treat such patients.

## 1. Introduction

Factor V (FV) plays crucial roles in the coagulation pathway as it interacts with coagulation factor X to form a complex that will ultimately activate prothrombin to convert it to thrombin. It also interacts with activated protein C (APC), which normally inactivates coagulation FV to prevent further clotting. Since FV is essential to the hemostatic pathway, disturbances to factor V, such as mutations in factor V gene, also known as factor V Leiden, and severe factor V deficiency, also known as parahemophilia, can cause disruptions in the equilibrium of the pathway. Factor V Leiden can decrease the rate of cleaving activated protein C thus rendering a hypercoagulable state, while FV deficiency can cause bleeding diathesis resulting in mild to severe bleeds despite factor V activity levels <1% for unknown reasons. Isolated mutations in F5 gene and combined deficiency in factors V and VIII, as seen in F5F8D, can cause a heterogenous bleeding phenotype. Factor V deficiency is extremely rare affecting 1 : 1,000,000 of the population; inhibitors to this condition can arise from exposure to bovine thrombin, autoimmune etiologies, and beta lactamase exposure and are exceedingly rare [1]. We describe a case report of our patient with severe factor V deficiency with inhibitor with moderate epistaxis and treatments used for hemostasis.

## 2. Case Presentation

A 77-year-old-female with a past medical history of an acquired factor V (FV) inhibitor presented with three days of slow but persistent epistaxis. Prior to presentation, she had two hospitalizations for bleeding. She was initially diagnosed with factor V deficiency with presence of an inhibitor initially in 2011 when she presented with an episode of excessive bleeding after tooth extraction. Her fibrinogen, platelets, vWF panel, and d-dimer were normal, hemoglobin at 6.9 mg/dL; PT was prolonged at 113 s (normally 9 to 12 seconds), PTT at 150 (normally 22 to 36 seconds), and did not correct after receiving total of 15 u PRBCs (packed red blood cells), 17 u FFP (Fresh Frozen Plasma), and vitamin K. Mixing study corrected PT; however PTT remained elevated. Individual factor assays yielded a factor V level <10% and later inhibitor titer at 15 Bethesda units (BU). Additional evaluation for lymphoproliferative disorders, paraproteinemias, and infectious workup was unremarkable. Her hospital course was complicated by a large retroperitoneal hemorrhage that was identified after her hemoglobin continued to trend down despite multiple transfusions. Her course was further complicated by the development of transfusion related acute lung injury (TRALI) that required intubation and was treated with steroids, NovoSeven, pheresis, and Rituxan. In 2013, the patient was admitted with a 8.7 × 3.8 × 21.0 cm right leg hematoma that developed immediately after an unspecified trauma to the area. She was treated with factor eight inhibitor bypassing activity (FEIBA), steroids, and Rituxan, but that hospital course was complicated by bilateral upper extremity deep vein thromboses.

On this admission, the patient's initial complete blood count (CBC) was unremarkable with a hemoglobin of 14.3 mg/dL, a hematocrit of 42.6, platelets of 255,000, and a white blood cell count of 10,000 with a normal differential. Her PTT was greater than 150, and she was found to have a PT of 108 and INR of 8.8 (normally 1). The mixing study was abnormal and did not correct with normal pooled plasma. The factor V activity was <1% with factor V inhibitor titers at 9.0 B.U. She was treated with 50 u/kg FEIBA every 12 hours and weekly Rituxan 375 mg/m^2^. An otolaryngologist then cauterized the source of epistaxis, which provided temporary hemostasis. By the ninth day of her hospitalization, the patient's hemoglobin had slowly dropped to 7.1 mg/dL so her FEIBA was increased to 75 u/kg every 8 hours and cyclophosphamide 100 mg daily was added for additional immunosuppression. At the time, the hemolytic workup, including lactate dehydrogenase, haptoglobin, serial hemoglobin and hematocrit, and a computed tomography (CT) scan of the abdomen/pelvis, was negative. On the fourteenth day of her hospitalization, her platelets were low at 28,000, and she subsequently developed hematuria with acute kidney injury. Her serum creatinine climbed to 7.5, and she became anuric. Her serum bilirubin increased to 2.8 mg/dL, with a predominant indirect bilirubin at 2.2 mg/dL. A repeat hemolytic workup revealed a serum haptoglobin that was less than 5 with an elevated LDH at 1837 IU/L. She continued to bleed and was thus shifted to NovoSeven 70 mcg/kg every 3 hours with renally adjusted cyclophosphamide. Her clinical presentation was thought to be consistent with a microangiopathic hemolytic anemia so she underwent hemodialysis and was treated with Soliris and prednisone 1 mg/kg for atypical HUS. Later, her ADAMTS13 returned within normal limits. Eventually, her epistaxis finally subsided as her PTT normalized. She completed 4 weekly Rituxan doses and received weekly Soliris for 3 weeks, and her factor V levels normalized to 54% activity with undetectable inhibitor titers. She has since been successfully weaned off hemodialysis, steroids, and cyclophosphamide, with her last factor V activity level at 114%.

## 3. Discussion

Factor V (FV) deficiency is typically associated with excessive bleeding after invasive procedures and mucosal tract bleeding. Acquired FV inhibitor is extremely rare with an estimated incidence of 1 in 10 million. Treatment is often derived from series of case reports. Most patients with FV inhibitor have specific risk factors such as being exposed to surgical procedures, topical bovine thrombin, antibiotics specifically of the beta lactam group, blood transfusions, cancers, and autoimmune disorders [1, 2]. FV deficiency secondary to an inhibitor can occur at any age and has a variable phenotype resulting in a mixed presentation of clinical symptoms and laboratory findings. Consequently, it is often difficult for physicians to easily make a diagnosis [3]. Some case reports document patients who may still have a bleeding tendency, despite residual plasma FV activity being present [4]. Our patient did have brief penicillin exposure prior to her dental work and received topical thrombin formulation from her dentist prior to her initial admission when she presented to our institution. Though she was not exposed to antibiotics or other risk factors that may predispose her to developing a FV inhibitor, an inhibitor was nonetheless detected. Furthermore, during her last hospitalization, she developed microangiopathic hemolysis. While there has been some evidence to show a relationship between FV Leiden and thrombotic microangiopathies [5], there has been no relationship found with FV deficiency or inhibitors. There is also some literature suggesting the development of microangiopathic hemolytic anemia with FEIBA replacement, namely, disseminated intravascular coagulopathy.

The treatment of symptomatic patients with factor V inhibitors is mainly anecdotal and has relied on immunosuppressive drugs, such as rituximab and corticosteroids with or without cyclophosphamide, to successfully suppress the autoantibody production. Our patient was treated successfully with these agents during each hospitalization; however, the regimen has not caused a full remission of the inhibitor. In terms of the prognosis of patients with acquired FV inhibitors, a comprehensive review of 76 reported cases was reported with a median age of 72 of patients [6]. The most common sites of bleeding were the gastrointestinal tract and genitourinary tract; however 9% of patients did have intracranial bleeding, 7% had a retroperitoneal bleed, and there was an overall 12% mortality attributed to the extensive bleeding [6]. Inhibitors typically remit after treatment or the removal of the triggering factor. The median time to remission was 6 weeks, ranging from 1 week to 29 months [1]. Our patient was consistent with these other presentations in that her FV inhibitor was undetectable and her FV levels were normal during her bimonthly follow-up appointments after discharge from the hospital. The unique aspect of this case, though, is the recurrence of the FV inhibitor every two years despite not having any other risk factors prior to presentation.
